# A Highly Sensitive Refractive Index Sensor Based on a V-Shaped Photonic Crystal Fiber with a High Refractive Index Range

**DOI:** 10.3390/s21113782

**Published:** 2021-05-29

**Authors:** Xin Yan, Rao Fu, Tonglei Cheng, Shuguang Li

**Affiliations:** State Key Laboratory of Synthetical Automation for Process Industries, College of Information Science and Engineering, Northeastern University, Shenyang 110819, China; 2000695@stu.neu.edu.cn (R.F.); chengtonglei@ise.neu.edu.cn (T.C.); lishuguang@ise.neu.edu.cn (S.L.)

**Keywords:** surface plasmon resonance, photonic crystal fiber, refractive index sensor, sensitivity

## Abstract

This paper proposes a highly sensitive surface plasmon resonance (SPR) refractive index sensor based on the photonic crystal fiber (PCF). The optical properties of the PCF are investigated by modulating the refractive index of a liquid analyte. The finite element method (FEM) is used to calculate and analyze the PCF structure. After optimization, the fiber can achieve high linearity of 0.9931 and an average refractive index sensitivity of up to 14,771.4 nm/RIU over a refractive index range from 1.47 to 1.52, with the maximum wavelength sensitivity of 18,000.5 nm/RIU. The proposed structure can be used in various sensing applications, including biological monitoring, environmental monitoring, and chemical production with the modification and analysis of the proposed structure.

## 1. Introduction

The waveguide characteristics and structural controllability of photonic crystal fibers (PCFs) [1] have led to the high popularity of the PCF-based surface plasmon resonance (SPR) devices. With the development of the SPR sensors, more of its peculiarity has been discovered and discussed. The SPR-based optical fiber sensor has the advantages of ultra-high sensitivity [2] and resolution [3], fast response speed [4], small size [5], and real-time detection capability [6], and they have been widely applied to the environmental monitoring, disease diagnosis, biological analysis [7], and many other fields [8,9,10,11]. Fiber SPR sensors generally have higher refractive index (RI) sensitivities than the RI sensors based on the fiber grating or interferometer, and thus have been widely applied to many fields [12]. Commonly, to increase the evanescent wave outside an optical fiber, a certain amount of or the entire optical fiber cladding is deployed on a traditional SPR optical fiber sensor [13].

In recent years, many studies on improving the sensors’ performances have been conducted. Liang et al. [14] proposed a D-shaped PCF-RI sensor with a different coating combination and added Graphene and Zinc Oxide above silver as a plasmonic exciton source material, thus achieving an average sensitivity of 6000 nm/RIU in the refractive index range of 1.37–1.41. This sensor has excellent stability and a good specialty for oxidation resistance, but its sensitivity is two times lower than the proposed sensor and its detection range is also small. Al Mahfuz et al. [15] designed a highly sensitive PCF-SPR biosensor having a maximum wavelength sensitivity of 12,000 nm/RIU in the measurement range from 1.33 to 1.40 for both gold and silver as plasmonic materials, this sensor has high sensitivity and the advantage of being easy to prepare. Wang et al. [16] proposed a refractive index sensor based on PCF with ultra-wide detection range from 1.29 to 1.49, which filled the central air holes with analyte and preformed the fiber with gold wires. This sensor has a wide detection range and stability, but to realize this fiber, the requirements of temperature, pressure and tension are very high. Yang et al. [17] presented a graphene-Au coated PCF sensor with a side-polish D-shaped plane, where graphene on gold helped to enhance the sensor’s sensitivity because it could stably adsorb biomolecules and increase the propagation constant of the surface plasmon polarization.

In this paper, a V-shaped highly-sensitive SPR-PCF refractive index sensor is proposed. The finite element method (FEM) is used to calculate and analyze the PCF structure [18]. A nano-level gold film is coated on the second layer of the proposed structure to excite the SPR, and an analyte liquid is infused in the second, third and fourth V-shaped layers. The simulation results show that the resonance wavelength of the loss curve follows a linear fitting rule [19] within the detecting range. After optimization, high sensitivity of up to 14,771.4 nm/RIU is obtained.

## 2. Proposed Structure Design and Working Principle

**Figure 1:**
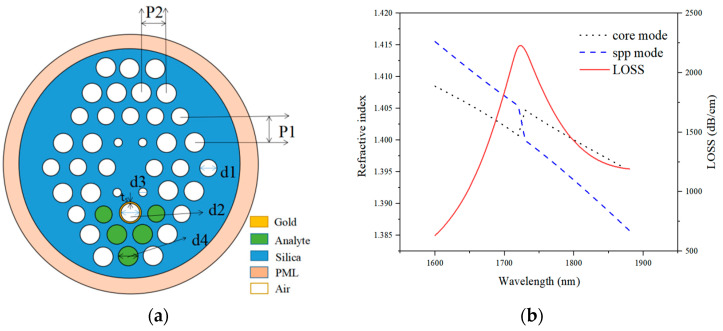
The cross-section view of the proposed SPR-PCF refractive index sensor (**a**). Real parts of the effective refractive indices of the core-guided and plasmonic modes and the core-guided mode loss under the gold layer thickness of 30 nm and RI of 1.50 (**b**).

The two-dimension (2D) cross-sectional view of the proposed SPR-PCF refractive index sensor is shown in Figure 1a, where it can be seen that the proposed structure has four layers of air holes. In the *y*-axis lattice, two adjacent air-holes center-to-center distance, which is defined as a lattice constant p_1_ is 1.7 μm, and in the *x*-axis lattice, the center-to-center distance is defined as a lattice constant p_2_, and p_2_ = 2.0 μm. The diameter d_1_ is 1.4 μm, and the inner diameter of the hole having the metal layer d_2_ is 1.6 μm. A thin gold layer is used as a plasmonic material, and its diameter t_g_ is 30 nm. The dielectric constant of gold is characterized by the Drude-Lorentz model, and the middle diameter denoted as d_3_ is 0.7 μm, while the diameter d_4_ is 1.7 μm. A V-shaped structure is used to fill in an analyte liquid, and to minimize the air holes between the fiber core and metal layer.

The proposed design uses the partial filling technology [20] to infuse refractive index sensitive liquid materials selectively into the second, third and fourth layers of air holes closest to the gold coated layer so that the surface plasmons can efficiently and effectively propagate through the metal-dielectric surface. First, the UV glue carried by the mental tip is aligned with the air holes of the target PCF. Second, a UV lamp is used to irradiate the end face of the PCF. Third, the end face of the PCF is inserted into the analyte, and a gas pump is used to pump it into the five air holes. Fourth, the first and second steps are reused to seal the five air holes filled with the analyte liquid. If all the air holes are filled with analyte liquid, then the fiber can’t have total reflection because of the high RI range of the analyte. The fused silica is selected in the proposed structure as the background material, and the diameter of the silica layer is 17 μm. To demonstrate the RI of fused silica, the well-known Sellmeier mathematical model is used, which is a distinct detail in the perfectly matched layer (PML) [21]; the thickness of PML is 1.0 μm, and the RI of the PML is set to be 0.03 higher than the RI of the substrate material—SiO_2_; the scattering boundary condition (SBC) is applied to the external section of the sensing analyte to avoid reflected echoes, and to absorb the energy of outward radiation. In the simulation, finer meshing elements were used to map the smaller air holes accurately. The computational domain took total triangular elements of 8992, edge elements of 1016, and vertex elements of 180. 

The peak loss caused by the core mode resonantly coupled to the metal defect mode is presented in Figure 1b, where the dispersion relation between the *y*-polarized core-guided mode and plasmonic mode for analyte RI of 1.50 are presented by the black-dot and blue-dot lines, respectively. The refractive index curves leap reversely when the loss curve reaches the peak. Therefore, the refractive index of a measured object can be obtained based on the position of the loss peak. Then, the sensitivity of the proposed structure under different peak shifts is analyzed using software the COMSOL Multiphysics 5.2a software on a computer.

**Figure 2:**
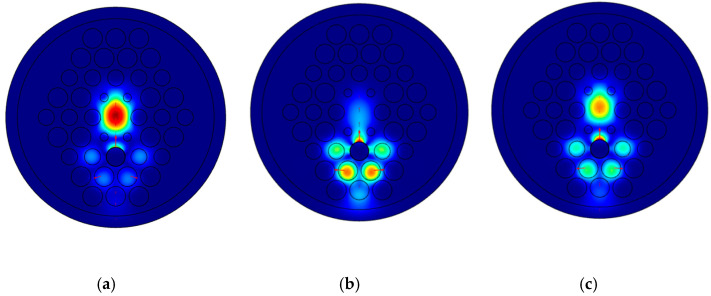
Core guided mode in the *y*-axis at λ = 1610 nm and RI = 1.50 (**a**), Plasmonic mode in the *y*-axis at λ = 1610 nm and RI = 1.50 (**b**), Coupling of the core mode and the plasmonic mode in the *y*-axis under the resonance wavelength (λ) of 1710 nm (**c**).

To distinguish the different conduction modes of this fiber, the energy distribution of coupling mode was analyzed at the wavelength in the range of 1600–1900 nm when the refractive index was 1.50, and the results are shown in Figure 2. The optical field distributions of the *y*-polarized core-guided mode, metal defect mode, and coupling of the core mode with the SPP mode are shown in Figure 2a–c, respectively. As the wavelength increases from 1600 nm to 1900 nm, the electric field strength of the core mode field changed from strong to weak and then to strong again. When the core mode and the metal defect mode were resonantly coupled, the core mode field energy was largely transferred and formed the resonant coupling mode.

The proposed fiber consists of fused silica. In the simulation process, the Sellmeier dispersion equation was used to set the scattering boundary conditions and finite element network, which was defined as [22]:

(1) $$n(\lambda )=\sqrt{1+\frac{B_{1}^{2}}{\lambda^{2}-C_{1}}+\frac{B_{2}^{2}}{\lambda^{2}-C_{2}}+\frac{B_{3}^{2}}{\lambda^{2}-C_{3}}}$$

where λ represented the wavelength of the free space in microns, and the other fitting constants were set as given in [23]. In this study, gold was used to excite the SPR. 

The dispersion of gold can be described by the Drude-Lorentz model [24] as follows:

(2) $$\varepsilon_{m}=\varepsilon_{\infty }-\frac{\omega_{D}^{2}}{\omega \left(\omega -{j\gamma }_{D}\right)}-\frac{\Delta \varepsilon \cdot \Omega_{L}^{2}}{\left(\omega^{2}-\Omega_{L}^{2}\right)-{j\Gamma }_{L}\cdot \omega }$$

where $\varepsilon_{\infty }$ = 5.9673 denotes the permittivity at infinite frequency, Δε = 1.09 is a weighting factor, and ω denotes the angular frequency of the guiding light, $\omega_{D}$ and $\gamma_{D\;}$ are the plasmon frequency and the damping frequency, and${\;\omega }_{D}/2\pi$ = 2113.6 THz and $\gamma_{D\;}/2\pi$ = 15.92 THz, respectively; $\Omega_{L}$ and ${\;\Gamma }_{L}$ represent the frequency and the spectral width of the Lorentz oscillator, and $\Omega_{L}/2\pi$ = 650.07 THz and ${\;\Gamma }_{L}/2\pi$ = 104.86 THz, respectively. The parameters of gold are given in [25].

In the calculation, a perfectly matching layer is introduced to absorb the radiation energy incident from different angles. By using the imaginary parts of effective refractive indices of the core and SPP modes, their confinement losses (L_C_) can be calculated by [26]:

(3) LC=20ln10·2πλ·Im[neff]×106(dB/cm)

where λ denotes the wavelength expressed in μm which is proportional to the Im[neff] that is the imaginary part of the effective refractive index. A large number of free electrons are present on the PCF coated metal surface and their free movements generate plasma waves near the metal surface. When the propagation constant of the incident light wave matches the propagation constant of the plasma wave on the metal surface, free-electron resonance in the metal film is caused. In the performance measurement, the focus was only on the *y*-polarized fundamental core mode since it had a sharper and higher loss peak in the propagation direction.

## 3. Simulation Result Analysis and Discussion

**Figure 3:**
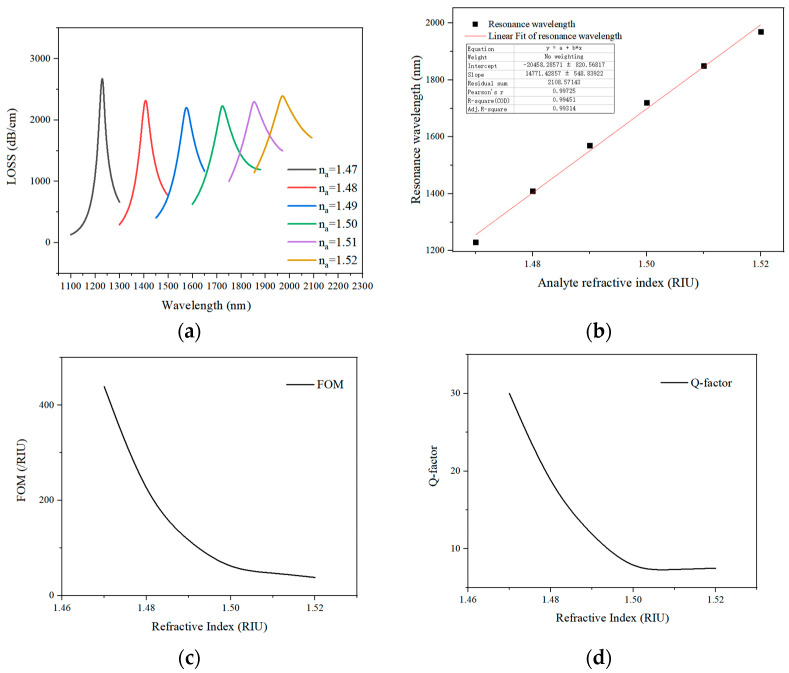
The confinement loss curve under different analyte refractive index values (**a**). The refractive index and the peak wavelength of the linearly-fitted analyte (**b**). The FOM profile for the designed structure (**c**). The corresponding Q-factor for RI range from 1.47 to 1.52 (**d**).

The sensing performance of the proposed structure was studied in the refractive index range from 1.470 to 1.520 with the step of 0.01. The relationship between the peak loss of the core mode versus the incident wavelength when the refractive index of the analyte increasing from 1.470 to 1.520 with the increment of 0.01 is displayed in Figure 3a. With the gradual increase in the refractive index, the resonance loss curve realized a significant red shift, and the loss value peak first gradually decreased at first and then increased. That was because the RI of SPP mode increased with the analyte’s RI, but the RI of the core mode remained unchanged, which caused the rise of the SPP mode RI curve and the red shift in the resonance wavelength. Since phase matching was satisfied between the core mode and the surface plasmon mode, the SPR was generated. The energy of the core mode was transferred to the metal surface to form a surface plasmon mode, so the core mode loss curve was generated. The increase in the refractive index of the liquid altered the phase-matching conditions. The loss matching wavelength gradually increased, and the resonance loss curve performed a significant red shift. As the refractive index of the liquid material had a high value, it was relatively prone to absorb the light. Therefore, the core loss of the fiber gradually increased with the RI of the analyte. The refractive index and peak wavelength of the linearly fitted analyte are presented in Figure 3b. Within the analyte RI range, the linear fitting result of the refractive index sensor could be expressed as *y* = 14,771.43*x* − 20,458.29, the linear fit was R^2^ = 0.99314, and the average sensitivity was 14,771.43 nm/RIU. 

By analyzing the loss curve, the PCF sensor was reckoned that it had good refractive index sensing performance over such a high measurement range, and the lowest loss value was 2410.8 dB/cm over the test range of 1.470–1.520. Large losses could shorten the fiber’s length, making the sensor small enough to be integrated into equipment for inspection. 

The wavelength sensitivity ${\;S}_{\lambda \;}\;$was calculated as follows [27]:

(4) $$S_{\lambda }\left(\mathrm{nm}/\mathrm{RIU}\right)=\frac{{\Delta \lambda }_{\mathrm{peak}}}{\Delta_{n_{a}}}$$

where ∆λ peak denoted the deviation between the adjacent peak wavelength shifts, and ∆n_a_ was the deviation of the analyte’s RI. 

As a factor in this paper, the figure of merit (FOM) on which the sensing enactment is being influenced is discussed. The FOM was calculated as follows [28]:

(5) $$\mathrm{FOM}=\frac{S_{\lambda }}{\mathrm{FWHM}}\;(/\mathrm{RIU})$$

where FWHM is the full width at half-maximum of the transmittance spectrum, and the FWHM of the loss curve are 41 nm, 79 nm, 135 nm, 242 nm, 252 nm and 263 nm corresponding to the RI range from 1.47 to 1.52.

An important factor of a wavelength filter is the capacity to give high wavelength selectivity, which represents a high-quality factor (Q) [29]:

(6) $$Q\;=\frac{\lambda_{\mathrm{res}}}{\mathrm{FWHM}}$$

where the $\lambda_{\mathrm{res}}$ is the resonance wavelength of the core mode and the SPP mode.

### 3.1. Discussion on Different Structural Parameter

#### 3.1.1. Discussion on d_1_ Optimal Value

**Figure 4:**
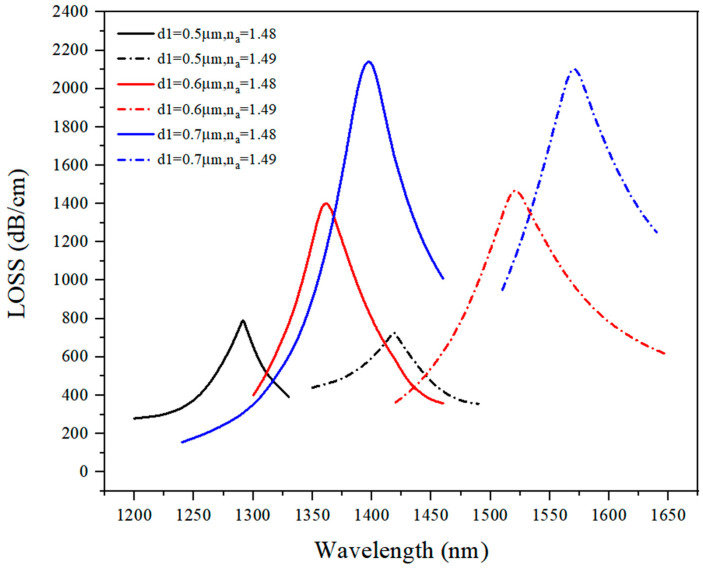
The loss spectrum (n_a_ = 1.48, n_a_ = 1.49) when d_1_ varied from 0.5 μm to 0.7 μm.

As shown in Figure 4, with the increase in the analyte RI, the loss spectrum made a red shift. When d_1_ = 0.5 μm, the loss curves corresponding to both n_a_ = 1.48 and n_a_ = 1.49 indicated wide bandwidth, and the loss curve had a lower peak value than d_1_ = 0.6 μm and d_1_ = 0.7 μm, so it could be concluded that 0.5 μm was not a good choice for the size of d_1_.

The analysis results showed that d_1_ = 0.7 μm represented a better option than the other two parameter values since it provided a higher peak value and a lower bandwidth. The reason for this result could be the increase in the value of d_1_, which increased the power transmitted between the gold layer and the analyte. Under a larger value of d_1_, the light energy could easily penetrate into the analyte liquid, so the loss peak value was higher. When d_1_ > 0.7 μm, the power of light would propagate more into the second layer of analyte-filled holes, and the core mode could not resonate with the SPP mode. After optimization, d_1_ = 0.7 μm was selected as an optimal value of d_1_.

#### 3.1.2. Discussion on d_2_ Optimal Value

**Figure 5:**
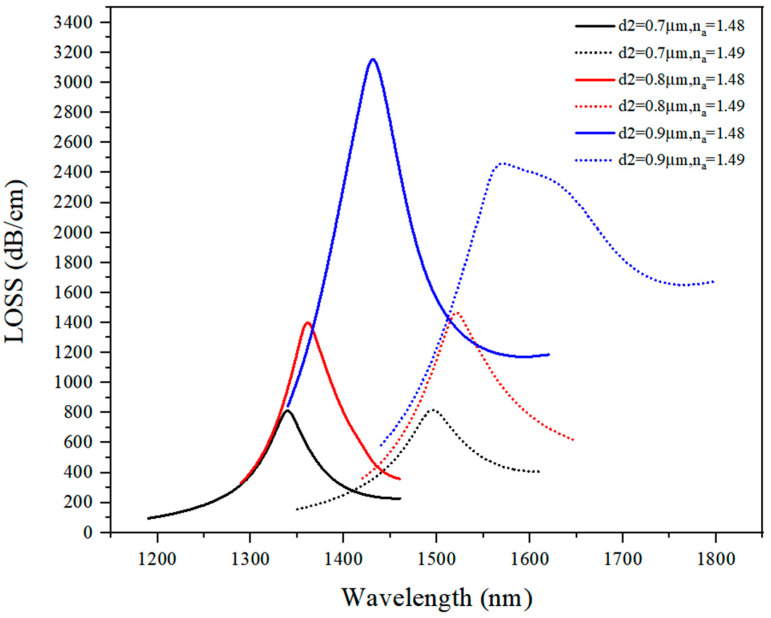
The loss spectrum (n_a_ = 1.48, n_a_ = 1.49) when d_2_ varied from 0.7 μm to 0.9 μm.

The loss spectrum when the value of d_2_ varied from 0.7 μm to 0.9 μm is presented in Figure 5, where it can be seen that the loss curve showed a red shift when the refractive index of the analyte increased.

As d_2_ increased, the loss peak value also increased, and the loss was in direct proportion to d_2_. When d_2_ = 0.9 μm (n_a_ = 1.48), the peak value reached 3200 dB/cm, but at n_a_ = 1.49, the loss curve showed certain distortions. Using the mode field distribution map in the COMSOL software, two different plasmon modes that realized the power transmission with the core mode were observed, but these two plasmon modes’ phase matching points did not overlap. Thus, d_2_ = 0.9 μm did not represent an optimal solution, and d_2_ = 0.8 μm denoted a better option than d_2_ = 0.7 μm. When d_2_ = 0.8 μm, the loss peak value was as high as 1400 dB/cm and the bandwidth was low. Based on the analysis results comparison, d_2_ = 0.8 μm was selected as an optimal value for d_2_.

#### 3.1.3. Discussion on d_3_ Optimal Value 

**Figure 6:**
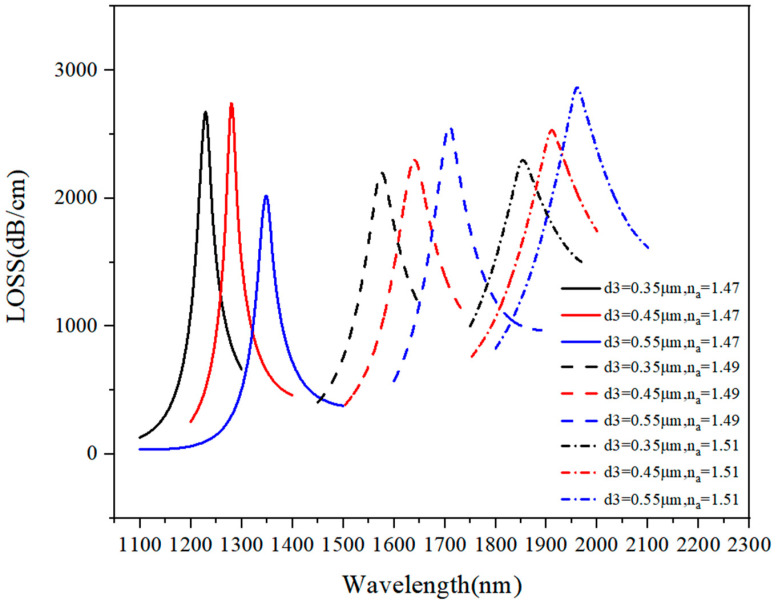
The loss spectrum (n_a_ = 1.47–1.51) when d_3_ varied from 0.35 μm to 0.55 μm.

The loss curve was also analyzed when d_3_ varied from 0.35 μm to 0.55 μm under n_a_ of 1.47–1.52, and the obtained results are presented in Figure 6.

When n_a_ = 1.47 and d_3_ = 0.55 μm, the core mode had incomplete coupling with the plasmon mode, so the loss curve had a low peak value. Besides, when d_3_ = 0.55 μm and d_3_ = 0.45 μm, the loss spectrum tended to have a higher loss peak and a lower bandwidth, but as d_3_ increased, the loss curve showed a red shift. The results given in Table 1, indicate that at d_3_ = 0.35 μm, the loss curve had a wider range (n_a_ = 1.47–1.52) than d_3_ = 0.45 μm and d_3_ = 0.55 μm, which had the range of n_a_ = 1.47–1.51; when n_a_ = 1.52, the phase matching points surpassed 2000 nm. Based on the results’ comparison, d_3_ = 0.35 μm was selected as an optimal value of d_3_.

#### 3.1.4. Discussion on d_4_ Optimal Value

**Figure 7:**
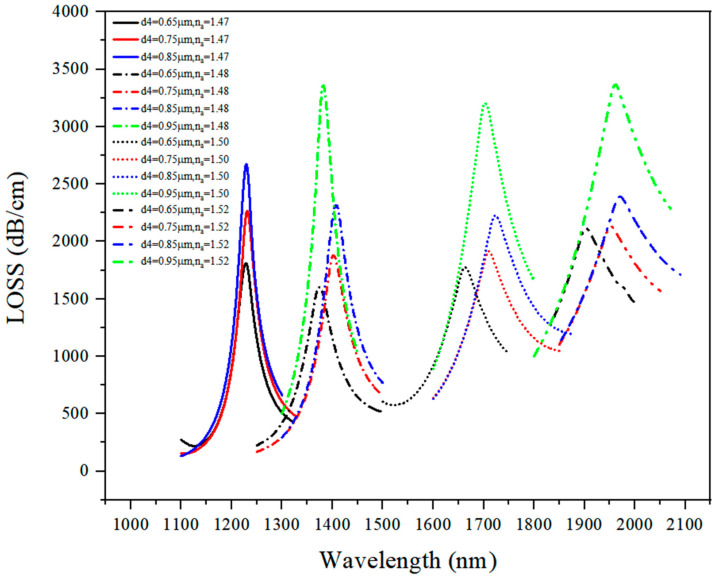
The loss spectrum (n_a_ = 1.47–1.52) when d_4_ varied from 0.65 μm to 0.95 μm.

The loss spectrum under n_a_ of 1.47–1.52 when the d_4_ varied from 0.65 μm to 0.95 μm is presented in Figure 7, and the numerical results are given in Table 2, the loss curve showed a red shift when the analyte RI increased. 

In contrast, when the value of d_4_ increased, there was no apparent shift in the loss curve. When d_4_ = 0.95 μm, the loss peak value reached a high value. Based on the results in Table 2, n_a_ = 1.47 was not in the sensing range, and after calculating an average sensitivity, it was found that the RI sensor had the best sensing performance when d_4_ = 0.85 μm. Therefore, d_4_ = 0.85 μm was selected as an optimal value of d_4_.

#### 3.1.5. Discussion on p_2_ Optimal Value

**Figure 8:**
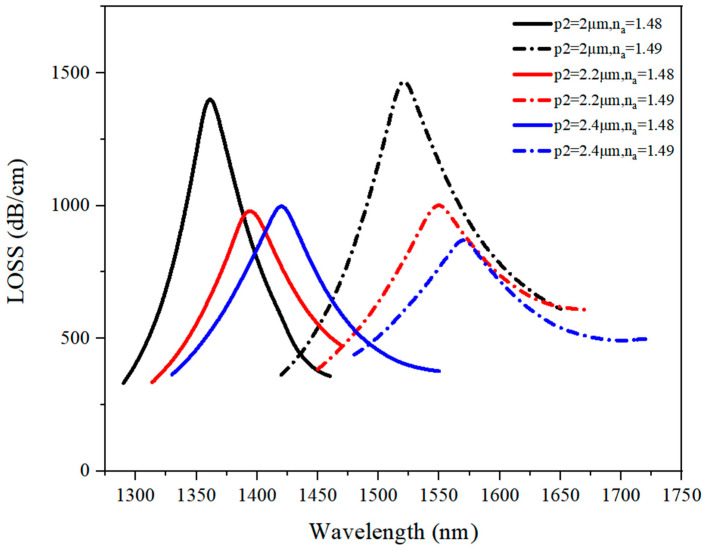
The loss spectrum (n_a_ = 1.48–1.49) when p_2_ varied from 2.0 μm to 2.4 μm.

The loss spectrum under n_a_ of 1.48–1.49 when p_2_ varied from 2.0 μm to 2.4 μm is presented in Figure 8.

As shown in Figure 8, the increase in both the analyte RI and the value of p_2_ caused the loss curve to make a red shift. When p_2_ = 2 μm, the loss value was higher than at p_2_ = 2.2 μm or p_2_ = 2.4 μm. Moreover, the loss curve had a lower bandwidth at p_2_ = 2 μm than at the other p_2_ values. The reason for this phenomenon was that an increase in p_2_ value reduced the power transmission between the gold layer and the analyte hole. As p_2_ increased, there was more space for the background material, fused silica, so a larger pitch reduced the surface plasmon resonance (SPR) effect.

#### 3.1.6. Discussion on t_g_ optimal value

**Figure 9:**
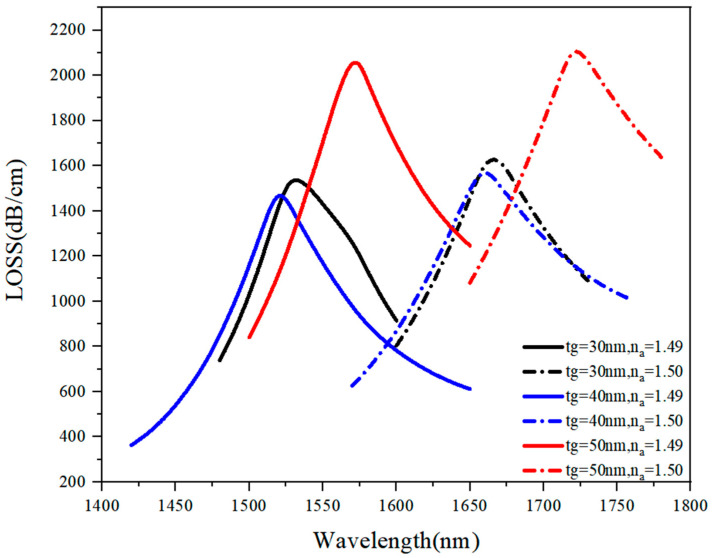
The loss spectrum (n_a_ = 1.49–1.50) when t_g_ varied from 30 nm to 50 nm.

The optimal value of t_g_ was also analyzed. First, all possible values of t_g_ from the range of 20 nm–60 nm were analyzed under n_a_ of 1.49–1.50, and the obtained results are presented in Figure 9. As shown in Figure 9, the loss curve had the highest loss peak value at t_g_ = 50 nm, but the bandwidth was relatively large, so t_g_ = 50 nm was discarded as a possible optimal value. Then, the loss curve was analyzed when t_g_ value was 20 nm, 30 nm, 40 nm, and 60 nm under n_a_ of 1.48–1.53, and the obtained results are presented in Table 3. As shown in Table 4, when t_g_ was 20 nm or 60 nm, the wavelength sensitivity was relatively low, i.e., about 12,000 nm/RIU, so only t_g_ values of 30 nm and 40 nm were left as possible optimal values; at both of these values, the average sensitivity was at the 15,000 nm/RIU-level. Therefore, the loss corresponding to t_g_ values of 30 nm and 40 nm were compared, and it was concluded that the loss curve corresponding to t_g_ value of 30 nm had lower bandwidth and higher peak value than that of t_g_ value of 40 nm, which was flatter. Consequently, a value of 30 nm was selected as an optimal t_g_ value.

### 3.2. Fabrication Tolerance

**Figure 10:**
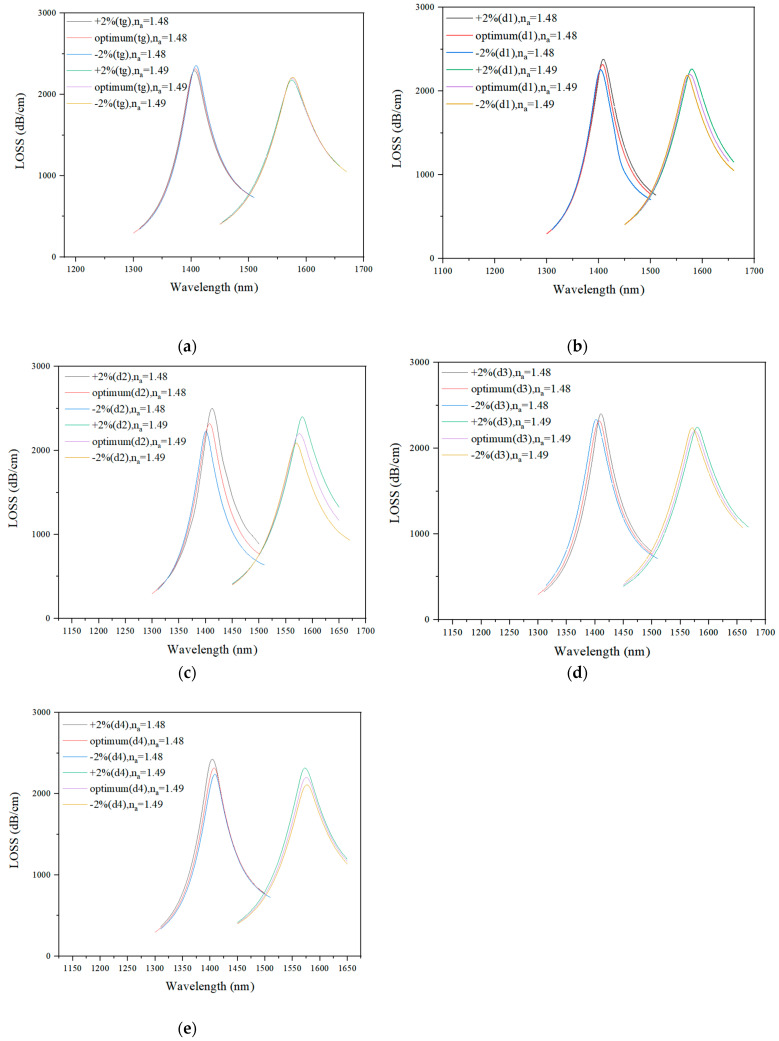
The loss spectrum when t_g_ varied by ±2% (n_a_ = 1.48, n_a_ = 1.49) (**a**). The loss spectrum when d_1_ varied by ±2% (n_av_= 1.48, n_a_ = 1.49) (**b**). The loss spectrum when d_2_ varied by ±2% (n_a_ = 1.48, n_a_ = 1.49) (**c**). The loss spectrum when d_3_ varied by ±2% (n_a_ = 1.48, n_a_ = 1.49) (**d**). The loss spectrum when d_4_ varied by ±2% (n_a_ = 1.48, n_a_ = 1.49) (**e**).

During the fabrication process, it is relatively unlikely to maintain the value of the proposed structure’s parameters. Usually, the variation of ±1% in the structural parameter value during the fabrication process has been considered acceptable. However, the proposed sensor had a fabrication tolerance of ±2%, as depicted in Figure 10. The proposed optimized sensor’s parameters were d_1_ = 0.7 μm, d_2_ = 0.8 μm, d_3_ = 0.35 μm, d_4_ = 0.85 μm, t_g_ = 30 nm, and p_2_ = 2 μm.

In Figure 10a,b,d, there are no obvious shifts in the loss curve, so changes in the values of t_g_, d_1_, and d_4_ did not have an evident effect on the loss value. As shown in Figure 10c, when the value of d_2_ increased, the loss curve had a tendency to perform a red shift, and the peak value also increased. Figure 10e shows that the change in the value of diameter d_4_ did not result in an obvious loss curve shift but affected and increased the peak value. In conclusion, the proposed sensor has an excellent fabrication tolerance ability.

## 4. Conclusions

In this paper, a simple PCF-based SPR sensor, which has a high sensitivity, is proposed and optimized. The analyte is deposited in a V-shape adjacent to the gold layer to minimize the fabrication complexity and improve the sensing performance. Under the optimized sensor parameters, the proposed sensor shows a maximum wavelength sensitivity of 18,005.2 nm/RIU and an average sensitivity of 14,771.42 nm/RIU in the analytes measurement range of 1.47 and 1.52. The fabrication tolerance investigation has shown that the sensor parameters vary by *±*2%, but these variations have no evident impacts on the sensing performance. Due to its simple structure and outstanding sensing characteristics, the proposed biosensor can be deployed in the liquid refractive index detection field.

## Figures and Tables

**Table 1:** The resonant wavelength (nm) under different RI values (n_a_ = 1.47–1.52) when d_3_ varied from 0.35 μm to 0.55 μm.

d_3_ (μm)	n_a_ (RIU)					
	1.47	1.48	1.49	1.50	1.51	1.52
0.35	1230.3	1410.1	1570.4	1720.1	1850.7	1970.9
0.45	1280.4	1470.0	1640.3	1780.2	1910.1	2020.6
0.55	1345.6	1540.5	1710.1	1850.2	1960.9	2060.4

**Table 2:** The resonant wavelength of different RI (n_a_ = 1.47–1.52) when d_4_ varied from 0.65 μm to 0.95 μm.

d_4_ (μm)	n_a_ (RIU)						Average Sensitivity (nm/RIU)
	1.47	1.48	1.49	1.50	1.51	1.52	
0.65	1230.1	1370.2	1520.8	1660.7	1790.1	1900.0	13,571.4
0.75	1230.2	1400.7	1570.1	1710.4	1840.4	1950.1	14,457.1
0.85	1230.1	1410.9	1570.5	1720.6	1850.5	1970.5	14,771.4
0.95		1380.8	1550.4	1700.1	1850.5	1960.6	14,600.0

**Table 3:** The resonant wavelength of different RI values (n_a_ = 1.47–1.53) under t_g_ of 20 nm, 30 nm, 40 nm, and 60 nm.

d_4_ (μm)	n_a_ (RIU)							Average Sensitivity (nm/RIU)
	1.47	1.48	1.49	1.50	1.51	1.52	1.53	
20		1420.4	1560.4	1690.3	1810.6	1910.9	2010.1	11,771.42
30	1230.1	1410.1	1570.2	1720.5	1850.4	1970.7		14,771.43
40	1205.3	1400.1	1570.1	1720.2	1850.3	1970.1		15,214.29
60		1350.5	1520.7	1660.4	1780.7	1890.0	1990.1	12,657.14

**Table 4:** Performance comparison of fiber optic sensors based on SPR.

Feature	RI Range (RIU)	Average Sensitivity (nm/RIU)	Maximum Sensitivity (nm/RIU)	Reference
D-Shaped coated with Graphene and Zinc Oxide	1.36–1.47	4485.7	6000	[11]
Graphene-Enhanced Liquid Refractive Index Sensor	1.3330–1.3688	2290	-	[5]
Wide-Range of Refractive Index Sensor	1.35–1.46	1931.03	-	[1]
Graphene-Au Coated Plasmon Resonance PCF Sensor	1.32–1.41	3900	4200	[14]
Highly sensitive photonic crystal fiber plasmonic biosensor	1.33–1.40	-	12,000	[12]
Proposed Sensor	1.47–1.52	14,771.4	18,000.5	

## Data Availability

No new data were created or analyzed in this study. Data sharing is not applicable to this article.
